# Removal of N-terminal tail changes the thermostability of the low-temperature-active exo-inulinase InuAGN25

**DOI:** 10.1080/21655979.2020.1809921

**Published:** 2020-08-30

**Authors:** Limei He, Rui Zhang, Jidong Shen, Ying Miao, Xianghua Tang, Qian Wu, Junpei Zhou, Zunxi Huang

**Affiliations:** a Engineering Research Center of Sustainable Development and Utilization of Biomass Energy, Ministry of Education, Yunnan Normal University, Kunming, People’s Republic of China; b College of Life Sciences, Yunnan Normal University, Kunming, People’s Republic of China; c Key Laboratory of Yunnan for Biomass Energy and Biotechnology of Environment, Yunnan, Kunming, People’s Republic of China

**Keywords:** Enzyme, biochemical property, terminus, structure, mechanism, mutagenesis

## Abstract

Exo-inulinases are members of the glycoside hydrolase family 32 and function by hydrolyzing inulin into fructose with yields up to 90–95%. The N-terminal tail contributes to enzyme thermotolerance, which plays an important role in enzyme applications. However, the role of N-terminal amino acid residues in the thermal performance and structural properties of exo-inulinases remains to be elucidated. In this study, three and six residues of the N-terminus starting from Gln23 of the exo-inulinase InuAGN25 were deleted and expressed in *Escherichia coli*. After digestion with human rhinovirus 3 C protease to remove the N-terminal amino acid fusion sequence that may affect the thermolability of enzymes, wild-type RfsMInuAGN25 and its mutants RfsMutNGln23Δ3 and RfsMutNGln23Δ6 were produced. Compared with RfsMInuAGN25, thermostability of RfsMutNGln23Δ3 was enhanced while that of RfsMutNGln23Δ6 was slightly reduced. Compared with the N-terminal structures of RfsMInuAGN25 and RfsMutNGln23Δ6, RfsMutNGln23Δ3 had a higher content of (1) the helix structure, (2) salt bridges (three of which were organized in a network), (3) cation–π interactions (one of which anchored the N-terminal tail). These structural properties may account for the improved thermostability of RfsMutNGln23Δ3. The study provides a better understanding of the N-terminus–function relationships that are useful for rational design of thermostability of exo-inulinases.

## Graphical Abstract

**Figure:**
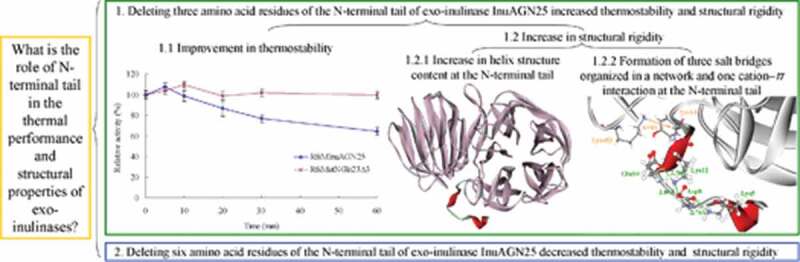

## 1. Introduction

Exo-inulinases are members of the glycoside hydrolase family (GH) 32 and function by hydrolyzing inulin into fructose with yields up to 90–95% [1]. The function has attracted much attention because the process needs only a single step with a high yield and inulin is the second most abundant storage polysaccharide in nature [1]. The product, fructose, is a common sweetening agent and fermentable sugar that is widely used in the food, beverage, and pharmaceutical industries.

Thermal performance is one of the critical properties for the biotechnological exploitation of enzymes. Enzymes adapted to high and low temperatures are both favorable depending on the processes of various industries [2]. When hydrolyzing inulin at low temperatures (lower than 25°C), the heating cost will be reduced [3]. However, to suit the industrial application, exo-inulinases are usually expected to have high activity and good stability at high temperatures because of the solubility problem of inulin at low temperatures [4].

The N-terminal and C-terminal regions contribute to the thermal performance of enzymes. For example, mutagenesis of N-terminal residues significantly improved the thermostability of *Penicillium janthinellum* GH 10 xylanase [5] and *Neocallimastix patriciarum* GH 11 xylanase [6]. The deletion of 85 N-terminal residues markedly decreased the activity and thermostability of the cold-adapted pullulanase from *Bacillus methanolicus* [7]. Zhou et al. successfully fused an inulin-binding module to the C-terminal region of exo-inulinase INU from *Kluyveromyces marxianus*, and the thermostability of the hybrid enzyme was enhanced [8].

The effects of N-terminal tail of exo-inulinases on thermal performance and structural properties have not been reported to date. Most mutagenesis studies for exo-inulinases have been aimed to reveal the catalytic residues, substrate-binding residues, and effects of conserved amino acid residues on exo-inulinase activity. For example, the conserved Asp189 from the Arg-Asp-Pro motif of *Aspergillus awamori* exo-inulinase was proven to provide hydrogen bonds important for substrate recognition according to the results of site-directed mutagenesis of Asp189 to asparagine [9]. Kuzuwa et al. reported that site-directed mutagenesis of the conserved amino acids Asp198, Arg388, Asp389, Glu440, Asp502, and Asp683 led to considerable activity loss of *Lactobacillus casei* exo-inulinase [10].

Previously, a novel exo-inulinase, InuAGN25, was identified as an atypical low-temperature-active enzyme with high activity at temperatures lower than 25°C and maximum activity at 40°C [3,11]. The aim of the study was to investigate the role of N-terminal amino acid residues in the thermal performance and structural properties of the exo-inulinase InuAGN25. The understanding of N-terminus–thermostability relationship is useful for rational design of thermostability of exo-inulinases.

## 2. Materials and methods

### 2.1. Chemicals, plasmids, and strains

All reagents (analytical grade) and materials were purchased from commercial suppliers, such as nickel-NTA agarose (Qiagen, Valencia, CA, USA), human rhinovirus 3 C protease (HRV 3 C protease; TaKaRa, Otsu, Japan), inulin from dahlia tubers (Sigma–Aldrich, St. Louis, MO, USA), isopropyl-β-d-1-thiogalactopyranoside (IPTG; Amresco, Solon, OH, USA), silica gel G plate (Haiyang, Qingdao, China), and the expression vector *p*ET-28a(+) (see Supplementary files) and strain *Escherichia coli* BL21 (DE3) (Synbio Technologies, Suzhou, China).

The exo-inulinase InuAGN25 and its encoding gene have been deposited in Genbank under accession numbers AGC01503 and JQ863108, respectively [3]. The gene was previously cloned from *Sphingobacterium* sp. GN25 and has been deposited in the China General Microbiological Culture Collection Center under CGMCC 1.10975 [3].

### 2.2. Enzyme expression in E. coli

The terminal amino acid residues derived from vectors, such as His tags, affect the thermolability of enzymes [12]. Previously, mature InuAGN25 (MInuAGN25) without the predicted signal peptide consisting of Met1 to Ala22 was heterologously expressed in *E. coli*; however, 18 amino acid residues containing a His tag derived from the *p*EASY-E1 vector were added at the N-terminus [3]. Therefore, the expression plasmid for recombinant InuAGN25 was reconstructed.

The expression plasmid reconstruction was performed using the *p*ET-28a(+) as vector ligated to the sequence encoding MInuAGN25 with the addition of an HRV 3 C protease recognition site, LEVLFQGP, encoded by 5′-CTGGAAGTTCTGTTCCAGGGGCCC-3′, at the N-terminus. The protease is commonly used to remove fusion sequences from recombinant low-temperature-active enzymes because its high specificity and high activity at low temperatures prevent the thermal denaturation of recombinant enzymes during the digestion process [13,14]. The recombinant sequence was ended with a stop codon of TAA to avoid the insertion of C-terminal amino acid residues derived from the vector. The linearized vector was amplified using the primer pair 5ʹ AGAGATAACACCACCACCACCACCACTG 3ʹ and 5ʹ CCTGGAACAGAACTTCCAGGAATTCGGATCCGCGACC 3ʹ with *p*ET-28a(+) as template and fast *Pfu* DNA polymerase. The insertion sequence was amplified using the primer pair 5ʹ TGGAAGTTCTGTTCCAGGGGCCCCAGACGGGACAGCATAAACAAG 3ʹ and 5ʹ GGTGGTGGTGTTATCTCTTAAATGCAGAAATACCGAT 3ʹ with genomic DNA of *Sphingobacterium* sp. GN25 as template and fast *Pfu* DNA polymerase. The linearized vector and insertion sequence were recombined using the ClonExpress II One Step Cloning Kit (Vazyme, Nanjing, China) according to the manufacturer’s instructions. The reconstruction plasmid, termed his_6_-hrv-MinuAGN25-p28, was sequenced by Tsingke (Beijing, China) to confirm the nucleic acid sequence.

One to ten amino acid residues of the N-terminal tail of MInuAGN25 (starting from Gln23 of InuAGN25) were deleted to generate mutants MutNGln23Δ1 to MutNGln23Δ10. The nucleic acid sequences encoding MutNGln23Δ1 to MutNGln23Δ10 were synthesized by Synbio Technologies. After digestion with *Eco*RI and *Sac*I, the sequences encoding MutNGln23Δ1 to MutNGln23Δ10 were individually inserted to the *p*ET-28a(+) vecotr using T4 DNA ligase. The nucleic acids of 10 mutant plasmids, termed his_6_-hrv-mutNGln23Δ1–10-p28, were confirmed by DNA sequencing (Tsingke, Beijing, China).

*E. coli* BL21 (DE3) was used as host transformed with wild-type his_6_-hrv-MinuAGN25-p28 and its mutants his_6_-hrv-mutNGln23Δ1–10-p28, and recombinant enzymes were induced and expressed inside the host cells as previously described for rXynAHJ3 [15].

### 2.3. Recombinant enzyme purification and fusion sequence removal

Recombinant enzymes inside the host cells were extracted and purified using immobilized metal affinity chromatography as performed previously for rInuAGN25 [3]. At this step, the recombinant enzymes, termed HHMInuAGN25 and HHMutNGln23Δ1 to HHMutNGln23Δ10, contained the fusion amino acid sequence MGSSHHHHHHSSGLVPRGSHMASMTGGQQMGRGSEFLEVLFQ (approximately 4.6 kDa) at their N-termini. To remove the amino acid fusion sequence, purified HHMInuAGN25, HHMutNGln23Δ3, and HHMutNGln23Δ6 were digested with HRV 3 C protease at 4°C for approximately 16 h. The digestion products were loaded onto nickel-NTA agarose gel columns to remove the cleaved amino acid fusion sequence and protease. The purified digestion products of HHMInuAGN25, HHMutNGln23Δ3, and HHMutNGln23Δ6 were designated as RfsMInuAGN25, RfsMutNGln23Δ3, and RfsMutNGln23Δ6, respectively. The elutions containing RfsMInuAGN25, RfsMutNGln23Δ3, and RfsMutNGln23Δ6 were dialyzed (molecular weight cutoff: 14 kDa) three times against 1 L of McIlvaine buffer (pH 7.0) to remove the elution buffer consisting of 20 mM Tris–HCl, 0.5 M NaCl, and 10% (w/v) glycerol.

### 2.4. Identification of enzymes

Cleavage of the amino acid fusion sequence was identified by sodium dodecyl sulfate-polyacrylamide gel electrophoresis (SDS-PAGE) using 5% polyacrylamide stacking gels and 12% resolving gels. The purity of the purified enzyme was also evaluated by SDS-PAGE.

### 2.5. Characterization of enzymes

The classic 3,5-dinitrosalicylic acid (DNS) method was employed to assay exo-inulinase activity with 0.5% (w/v) inulin as the substrate using half of the reaction system described previously [3]. Briefly, the reaction system containing 50 μl of enzyme solution and 450 μl of substrate solution was incubated in a water bath for 10 min, and then, the reaction was stopped by 750 μl of DNS reagent for measurement of absorption at 540 nm. The amount of enzyme releasing 1 μmol of fructose per minute was defined as one unit of activity.

The initial evaluation of thermostability change was determined in triplicate using crude enzymes. After incubating the crude enzyme at 60°C for 5 min without inulin, the thermostability of the enzyme was determined by measuring the residual activity at 37°C and pH 6.0 McIlvaine buffer.

The thermostabilities of purified RfsMInuAGN25, RfsMutNGln23Δ3, and RfsMutNGln23Δ6 were determined by measuring the residual activity at 37°C and pH 6.0 McIlvaine buffer after incubating these enzymes at 55°C for 5–30 min without inulin.

The enzymatic properties of purified RfsMInuAGN25 and RfsMutNGln23Δ3 were further characterized. The optimal pH of purified RfsMInuAGN25 and RfsMutNGln23Δ3 were determined at 37°C using inulin as the substrate dissolved in McIlvaine buffer. pH stability experiments were performed by measuring the residual activity at 37°C and pH 6.0 after the incubation of purified RfsMInuAGN25 and RfsMutNGln23Δ3 at 20°C for 1 h without a substrate in the pH range of 3.0 to 11.0 (pH 3.0–8.0 McIlvaine buffer and pH 9.0–11.0 0.1 M glycine–NaOH buffer). The optimal temperatures of purified RfsMInuAGN25 and RfsMutNGln23Δ3 were determined in pH 6.0 McIlvaine buffer. The thermostabilities of purified RfsMInuAGN25 and RfsMutNGln23Δ3 were determined by measuring the residual activity at 37°C and pH 6.0 McIlvaine buffer after incubating these enzymes at 45°C and 50°C for 5–60 min without inulin. Because GH 32 includes exo-inulinases and endo-inulinases, the modes of action of RfsMInuAGN25 and RfsMutNGln23Δ3 were investigated with 0.5% inulin (w/v) as the substrate hydrolyzed at 37°C, pH 6.0 for 4 h. The hydrolysis products were visualized using thin-layer chromatography (TLC) as described previously [3].

### 2.6. Structure analysis of enzymes

Structural analyses were performed to determine the factors that mainly cause the thermostability change. The tertiary structures of RfsMInuAGN25, RfsMutNGln23Δ3, and RfsMutNGln23Δ6 were modeled with I-TASSER [16], using the C-score and estimated TM-score to evaluate model quality. Salt bridges with an oxygen-nitrogen distance cutoff of 3.2 Å were predicted using VMD [17]. The energetically significant cation-π interactions (distance cutoff: 6.0 Å) within enzymes were predicted using CaPTURE [18].

## 3. Results

### 3.1. Enzyme expression in E. coli

The expression plasmids his_6_-hrv-MinuAGN25-p28 and his_6_-hrv-mutNGln23Δ1–10-p28 were successfully constructed with the addition of an HRV 3 C protease recognition site at the N-termini of the recombinant enzymes. *E. coli* BL21 (DE3) was successfully transformed with these plasmids for recombinant enzyme expression.

After induction, the recombinant enzymes were expressed inside the host cells. Crude recombinant enzymes were extracted after the cells were disrupted by sonication on ice. The removal of cell debris from crude enzyme solutions was carried out by centrifugation. The inulinase activities of these crude enzymes, except HHMutNGln23Δ5, were detected.

### 3.2. Thermostabilities of the crude recombinant enzymes

Crude HHMutNGln23Δ3 was stable at 60°C for 5 min, while crude HHMInuAGN25 and other mutants lost at least 65% activity (Figure S1). Although these data were not sufficiently accurate to reflect enzyme thermostability because of the use of crude enzymes, the results indicated that the thermostability of HHMutNGln23Δ3 was enhanced. The thermostabilities of other mutants seemed similar. As such, two mutants were selected for further study, HHMutNGln23Δ3 with enhanced thermostability and HHMutNGln23Δ6 representing mutants without significant thermostability change.

### 3.3. Recombinant enzyme purification and fusion sequence removal

**Figure 1.**
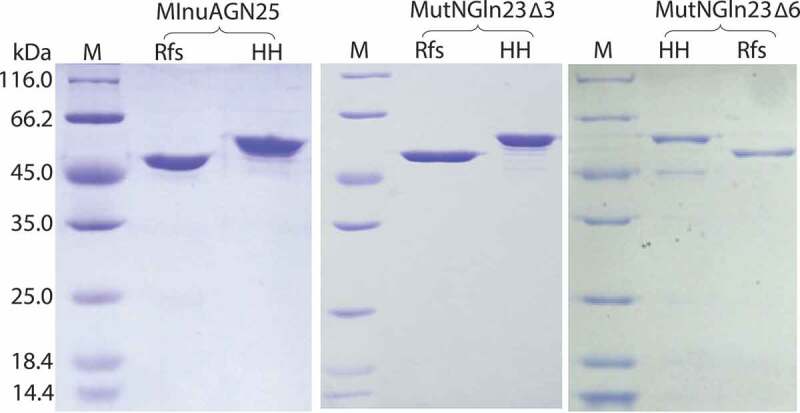
SDS-PAGE analysis. Lanes: M, protein molecular weight marker; HH, purified recombinant enzymes with a His_6_ tag and HRV 3 C protease recognition site at their N-termini; Rfs, purified enzymes without an amino acid fusion sequence at their N-termini.

Crude HHMInuAGN25, HHMutNGln23Δ3, and HHMutNGln23Δ6 were loaded onto nickel-NTA agarose gel columns for purification. The purified elutions were digested with HRV 3 C protease, then the fused amino acid sequence showing a molecular weight of approximately 4.6 kDa was cleaved (Figure 1). After digestion, the reaction products were purified with nickel-NTA agarose gel columns again to separate RfsMInuAGN25, RfsMutNGln23Δ3, and RfsMutNGln23Δ6 from the cleaved fusion sequence and protease. Finally, purified RfsMInuAGN25, RfsMutNGln23Δ3, and RfsMutNGln23Δ6 were successfully obtained (Figure 1).

### 3.4. Thermostabilities of the purified enzymes

**Figure 2.**
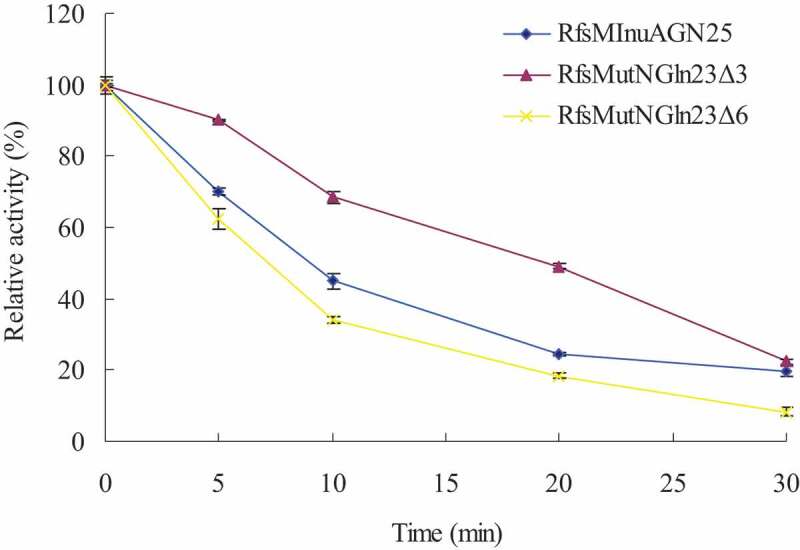
Thermostabilities of purified RfsMInuAGN25, RfsMutNGln23Δ3, and RfsMutNGln23Δ6. The thermostabilities of the enzymes were determined by incubating the purified enzymes at 55°C for 5–30 min without inulin. The error bars represent the means ± SD (*n* = 3).

After removing the fusion sequence, thermostability analysis indicated that the half-lives of RfsMInuAGN25, RfsMutNGln23Δ3, and RfsMutNGln23Δ6 at 55°C were approximately 10 min, 20 min, and 7 min, respectively (Figure 2). These results revealed that deletion of three amino acid residues of the N-terminal tail enhanced thermostability while deletion of six amino acid residues of the N-terminal tail slightly reduced thermostability.

### 3.5. Further characterization of RfsMInuAGN25 and RfsMutNGln23Δ3

**Figure 3.**
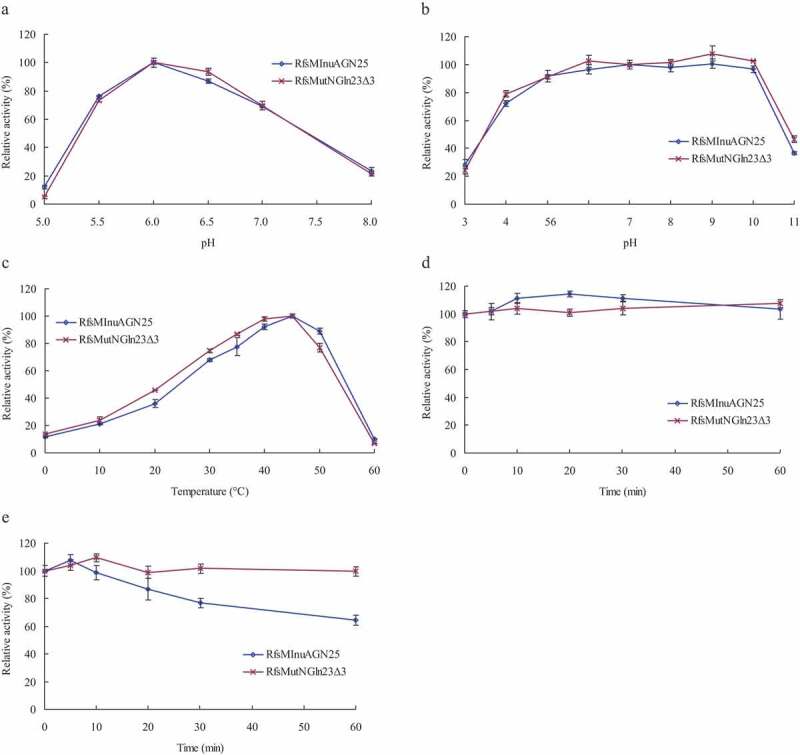
Effects of pH and temperatures on purified RfsMInuAGN25 and RfsMutNGln23Δ3. (a) pH-dependent activity. (b) pH-dependent stability. (c) Temperature-dependent activity. (d) Thermostability at 45°C. (e) Thermostability at 50°C. The error bars represent the means ± SD (*n* = 3).

Both RfsMInuAGN25 and RfsMutNGln23Δ3 showed the highest activity at pH 6.0 and good stability at the pH range of 4.0 to 10.0. The pH-dependent activity and stability curves of RfsMInuAGN25 and RfsMutNGln23Δ3 were almost the same (Figure 3a,b).

Both RfsMInuAGN25 and RfsMutNGln23Δ3 showed the highest activity at 45°C and good stability at the temperature of 45°C, with similar temperature-dependent activity and stability curves (Figure 3c,d). RfsMutNGln23Δ3 did not lose activity when it was incubated at 50°C for 5–60 min. However, when incubated at 50°C for 10–60 min, the activity of RfsMInuAGN25 was decreased from 99% to 64% (Figure 3e).

Both RfsMInuAGN25 and RfsMutNGln23Δ3 exhibited exo-inulinase characteristics because the enzymes hydrolyzed inulin to produce fructose (Figure S2).

### 3.6. Structural changes between RfsMInuAGN25 and its mutants

**Figure 4.**
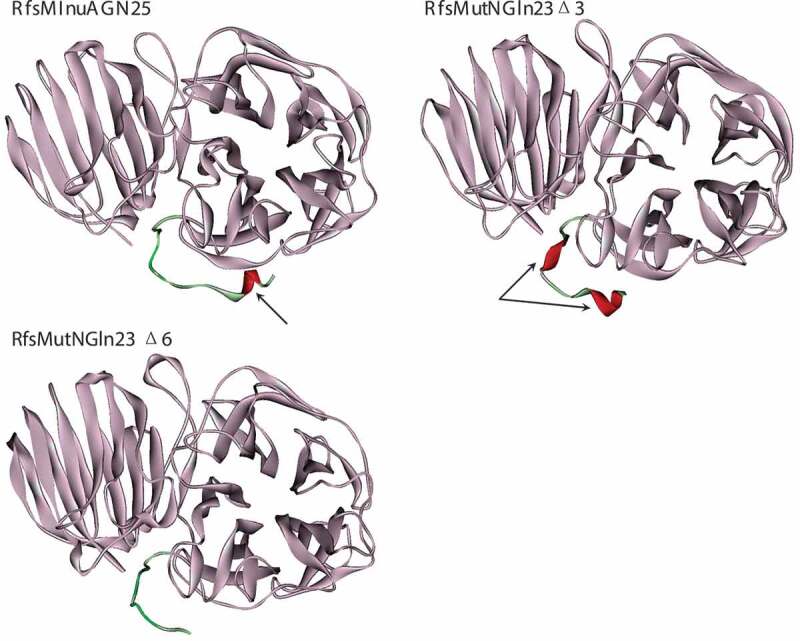
Tertiary structures of RfsMInuAGN25 and its mutants. The arrows indicate the changes in secondary structures.

Structural models of RfsMInuAGN25, RfsMutNGln23Δ3, and RfsMutNGln23Δ6 were successfully constructed (Figure 4). The C-scores for RfsMInuAGN25, RfsMutNGln23Δ3, and RfsMutNGln23Δ6 models were 1.15, 0.98, and 1.06, respectively. The estimated TM-scores for RfsMInuAGN25, RfsMutNGln23Δ3, and RfsMutNGln23Δ6 models were 0.87, 0.85, and 0.86, respectively. The C-scores and estimated TM-scores indicate that these models have high confidence and a correct topology.

A huge difference in the secondary structure was observed at the N-termini of these models (Figure 4). The amino acid residues of the N-terminal tail of RfsMInuAGN25 formed an α-helix plus a loop. The deletion of three amino acid residues of the N-terminal tail led to the formation of an α-helix–loop–3_10_-helix structure. When six amino acid residues of the N-terminal tail were deleted, only a loop was observed.

The deletion of residues of the N-terminal tails changed the amino acid interactions within RfsMInuAGN25, RfsMutNGln23Δ3, and RfsMutNGln23Δ6. Notably, the salt bridges formed by the N-terminal amino acid residues of RfsMInuAGN25, RfsMutNGln23Δ3, and RfsMutNGln23Δ6 were much different.

**Figure 5.**
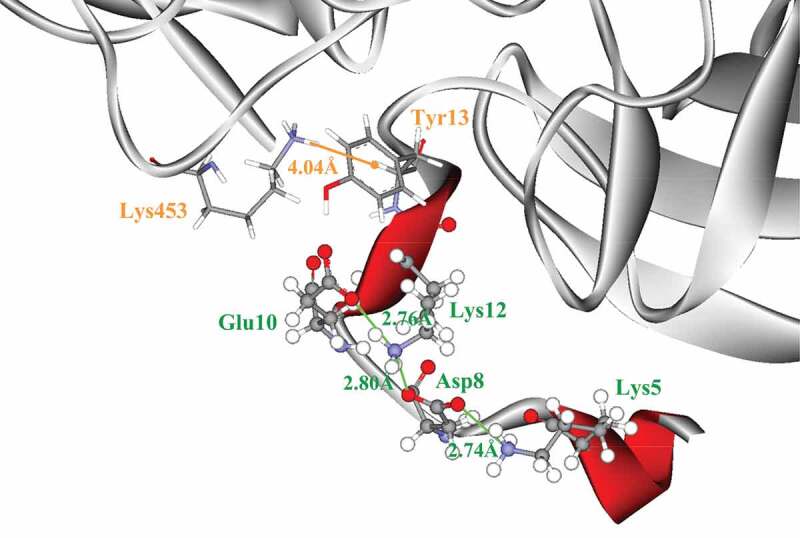
Salt bridge network (green) and energetically significant cation-*π* interaction (orange) related to the N-terminal tail of RfsMutNGln23Δ3. The amino acid residues involved in salt bridges are shown in ball-and-stick form. The amino acid residues involved in the cation-*π* interaction are shown in stick form.

The salt bridges of RfsMInuAGN25, RfsMutNGln23Δ3, and RfsMutNGln23Δ6 numbered 43, 47, and 43, respectively, in total (Table 1). Salt bridges related to the first 100 amino acid residues of the N-termini of RfsMInuAGN25, RfsMutNGln23Δ3, and RfsMutNGln23Δ6 numbered 9, 12, and 9, respectively. These results revealed that the deletion of three amino acid residues of the N-terminal tail led to an increased number of salt bridges, especially N-terminal salt bridges. Further analysis indicated that the RfsMutNGln23Δ3 structure had a salt bridge network, Lys5–Asp8–Lys12–Glu10, which was formed by three salt bridges with four amino acid residues of the N-terminal tail (Table 1; Figure 5). However, the effect was not observed for the deletion of six amino acid residues of the N-terminal tail.

**Table 1.** Salt bridges from RfsMInuAGN25 and its mutants.

Enzyme	Total number	Salt bridges related to the first 100 amino acid residues of the N-terminus
RfsMInuAGN25	43	9: Lys8–Glu73, Asp11–Arg17, Glu13–Lys456, Lys15–Glu404, Asp39–Lys136, Asp67–His70, Glu73–Lys75, Asp81–Lys142, Lys95–Asp96
RfsMutNGln23Δ3	47	12: Lys5–Asp8, Asp8–Lys12, Glu10–Lys12, Glu11–Arg14, Asp36–Lys133, Glu48–Lys49, Lys63–Asp64, Asp64–His67, Glu70–Lys72, Asp78–Lys139, Asp78–Lys141, Lys92–Asp93
RfsMutNGln23Δ6	43	9: Glu7–Lys450, Glu8–Arg11, Glu8–Lys296, Lys9–Glu398, Asp33–Lys130, Lys60–Asp61, Asp75–His106, Asp75–Lys136, Lys89–Asp90

The numbers of energetically significant cation-*π* interactions in the structures of RfsMInuAGN25, RfsMutNGln23Δ3, and RfsMutNGln23Δ6 were 6, 7, and 6, respectively (Table 2). The cation-*π* interaction formed by Tyr13 and Lys453 anchored the N-terminal tail of RfsMutNGln23Δ3 (Figure 5).

**Table 2.** Energetically significant cation-*π* interactions.

Enzyme	Cation-*π* interactions
RfsMInuAGN25	6: Trp28–Arg304, Arg120–Trp155, Lys317–Phe322, Phe352–Arg462, Arg399–Phe411, Arg419–Phe428,
RfsMutNGln23Δ3	7: Tyr13–Lys453, Lys92–Trp161, Phe154–Lys191, Lys314–Phe319, Phe349–Arg459, Arg396–Phe408, Arg416–Phe425
RfsMutNGln23Δ6	6: Lys9–Tyr10, Lys89–Trp158, Lys256–Tyr317, Tyr382–Arg413, Arg393–Phe405, Arg393–Phe446

## 4. Discussion

Mutagenesis reports on the thermal performance of exo-inulinases are scarce. Deletion of the Ω-loop fragment, ^74^Tyr-Gly-Ser-Asp-Val-Thr^79^, from *Aspergillus niger* exo-inulinase resulted in thermostability loss at 60°C [19]. Substituting the histidine from ^189^Ala-Glu-Leu-His^192^ of the *A. niger* exo-inulinase with alanine resulted in thermostability loss at 60°C [20]. To enhance thermostability, *K. marxianus* exo-inulinase was fused to the inulin-binding module from the N-terminal region of *Bacillus macerans* cycloinulinooligosaccharide fructanotransferase [8]. Ma et al. reported that the thermostability of *Kluyveromyces cicerisporus* exo-inulinase was changed when five N-glycosylation sites (Asn362, Asn370, Asn399, Asn467, and Asn526) at the C-terminus were substituted with glutamines [21]. This study firstly showed that thermostability of exo-inulinases were affected by N-terminal tail.

Results of thermostability indicate that shortening the N-terminal tail can enhance the thermostability of exo-inulinases, but not always. Some of previous studies [22,23] showed similar results to this study. Deletion mutants of Val3, Asn7, and Gln11, as well as the triplet mutant of GH 26 mannanase Man1312, exhibited a thermostability increase, while deletion mutants of Val6 and Ala10 exhibited a thermostability decrease [22]. Sequential deletion of N-terminal residues from yeast iso-1-cytochrome-*c* also showed different effects: deletion of two residues of the N-terminus increased thermostability, while deletion of 1, 3, 4, and 5 residues of the N-terminus decreased thermostability [23].

Thermophilic enzymes have a higher content of helix structure than mesophilic homologs [24,25]. For example, the triplet deletion mutant of Val3, Asn7, and Gln11 of the GH 26 mannanase Man1312 exhibited a higher content of helix structure and better thermostability than the wild-type enzyme [22]. Additionally, the *A. niger* exo-inulinase exhibited a higher content of helix structure and better thermostability than the single-site mutant His192Ala [20]. Thus, a helix at the N-terminus may contribute to the thermostability of exo-inulinase RfsMInuAGN25 and its mutant RfsMutNGln23Δ3.

Salt bridges are believed to increase global or local rigidity of structure, thus improving protein thermostability [25,26]. In most cases, the number of salt bridges increases with improved thermostability of the protein [25,26]. Salt bridges organized in networks contribute more positive effects on thermostability than an equivalent number of isolated salt bridges [24]. A salt bridge network, Arg259–Asp17–Arg12–Asp183, is formed by three salt bridges with four amino acid residues in the structure of the thermostable subtilisin-like proteinase aqualysin I [27]. Asp17 and Arg12 are at the N-terminal tail of aqualysin I. When Asp17 is substituted with Asn, the salt bridge network is broken, leading to a remarkable effect on thermostability loss [27]. However, isolated salt bridges do not have a remarkable effect on the thermostability of aqualysin I [27]. Similar to the aqualysin I structure, the RfsMutNGln23Δ3 structure has a salt bridge network, Lys5–Asp8–Lys12–Glu10, which is also formed by three salt bridges with four amino acid residues of the N-terminal tail. Therefore, the increased number of salt bridges organized in a network enhances the local rigidity of the RfsMutNGln23Δ3 structure and improve the thermostability.

Cation–*π* interactions can become more stable at higher temperatures, and then they contribute to a larger stabilizing effect close to enzyme melting temperatures [26]. Tu et al. reported that introducing cation–*π* interactions to loops increased the thermostability of the GH28 endo-polygalacturonase from *Penicillium* sp. CGMCC 1669 [28]. Furthermore, the N-terminal tail is usually one of the regions that unfold first during thermal denaturation [24]. One strategy to stabilize the N-terminal tail is to anchor it with amino acid residues from the C-terminus [24]. In this study, the increased cation-*π* interaction formed by Tyr13 and Lys453 may increase the rigidity of loops and anchor the N-terminal tail of RfsMutNGln23Δ3.

## 5. Conclusions

The aim of the study was to figure out whether and how N-terminal tails affect the thermal performance and structural properties of exo-inulinases. The results revealed that shortening the N-terminal tail can enhance and reduce the thermostability of exo-inulinases, as well as change their structural properties. Deletion of three amino acid residues of the N-terminal tail enhanced the thermostability because of the increased number of N-terminal salt bridges and cation–π interactions, as well as enhanced the N-terminal rigidity contributed by the helix structure, one salt bridge network, and one cation–π interaction anchoring the N-terminal tail. These findings provide a better understanding of the N-terminus–function relationships that are useful for rational design of thermostability of exo-inulinases.

## Supplementary Material

Supplemental Material

## Abbreviations

GH: Glycoside hydrolase family; HRV: Human rhinovirus; IPTG: Isopropyl-β-d-1-thiogalactopyranoside; SDS-PAGE: Sodium dodecyl sulfate-polyacrylamide gel electrophoresis; DNS: 3,5-Dinitrosalicylic acid; TLC: Thin-layer chromatography; InuAGN25: Exo-inulinase from *Sphingobacterium* sp. GN25; MInuAGN25: Mature InuAGN25 without the predicted signal peptide consisting of Met1 to Ala22; rInuAGN25: Recombinant MInuAGN25 expressed in *E. coli* using *p*EASY-E1 as vector; HHMInuAGN25: Recombinant MInuAGN25 with amino acid fusion sequence, MGSSHHHHHHSSGLVPRGSHMASMTGGQQMGRGSEFLEVLFQ (containing a His_6_ tag and HRV 3C protease recognition site) at the N-terminus, expressed in *E. coli* using *p*ET-28a(+) as vector; RfsMInuAGN25: Removal of amino acid fusion sequence from HHMInuAGN25 by HRV 3C protease; MutNGln23Δ1 to MutNGln23Δ10: Deletion of one to ten amino acid residues of the N-terminal tail of MInuAGN25 (starting from Gln23 of InuAGN25); HHMutNGln23Δ1 to HHMutNGln23Δ10: Recombinant MutNGln23Δ1 to MutNGln23Δ10 with amino acid fusion sequence, MGSSHHHHHHSSGLVPRGSHMASMTGGQQMGRGSEFLEVLFQ (containing a His_6_ tag and HRV 3C protease recognition site) at their N-termini, expressed in *E. coli* using *p*ET-28a(+) as vector; RfsMutNGln23Δ3 and RfsMutNGln23Δ6: Removal of amino acid fusion sequence from HHMutNGln23Δ3 and HHMutNGln23Δ6 by HRV 3C protease; his_6_-hrv-MinuAGN25-p28: Plasmid constructed in this study to express HHMInuAGN25 using *p*ET-28a(+) as vector; his_6_-hrv-mutNGln23Δ1–10-p28: Plasmids constructed in this study to express HHMutNGln23Δ1 to HHMutNGln23Δ10 using *p*ET-28a(+) as vector.

## Highlights

> Role of N-terminal tail in thermostability of exo-inulinase was first revealed.

> Role of N-terminal tail in structural rigidity of exo-inulinase was first revealed.

> Deleting three and six residues of the N-terminal tail changed thermostability.

> Deleting three and six residues of the N-terminal tail changed N-terminal rigidity.

> Helix structure, salt bridges, and cation–π interactions affected thermostability.
